# Prescription of renin‐angiotensin‐aldosterone system inhibitors (RAASi) and its determinants in patients with advanced CKD under nephrologist care

**DOI:** 10.1111/jch.13563

**Published:** 2019-06-06

**Authors:** Roberto Pecoits‐Filho, Danilo Fliser, Charlotte Tu, Jarcy Zee, Brian Bieber, Michelle M. Y. Wong, Friedrich Port, Christian Combe, Antonio A. Lopes, Helmut Reichel, Ichiei Narita, Benedicte Stengel, Bruce M. Robinson, Ziad Massy, CKDopps Investigators, Christian Duttlinger, Johannes Duttlinger, Gerhard Lonnemann, Takashi Wada, Kunihiro Yamagata, Ron Pisoni, Viviane Calice da Silva, Ricardo Sesso, Elodie Speyer, Koichi Asahi, Junichi Hoshino, Rachel Perlman, Nidhi Sukul, Eric Young

**Affiliations:** ^1^ School of Medicine Pontificia Universidade Catolica do Parana Curitiba Brazil; ^2^ Arbor Research Collaborative for Health Ann Arbor Michigan; ^3^ Department of Internal Medicine IV Saarland University Medical Center Homburg Germany; ^4^ Department of Medicine University of British Columbia Vancouver Canada; ^5^ Department of Internal Medicine, Michigan Medicine, and Department of Epidemiology, School of Public Health University of Michigan Ann Arbor Michigan; ^6^ Service de Néphrologie Transplantation Dialyse Centre Hospitalier Universitaire de Bordeaux Bordeaux France; ^7^ Faculdade de Medicina da Bahia School of Medicine Universidade Federal da Bahia Brazil; ^8^ Nephrological Center Villingen‐Schwenningen Germany; ^9^ Division of Clinical Nephrology and Rheumatology Niigata University Graduate School of Medical and Dental Science Niigata Japan; ^10^ CESP, Center for Research in Epidemiology and Population Health University Paris‐Saclay, University Paris‐Sud, UVSQ Villejuif France; ^11^ Division of Nephrology Ambroise Paré University Hospital, APHP Boulogne Billancourt/Paris France

**Keywords:** albuminuria, chronic kidney disease, diabetes, heart failure, renin‐angiotensin‐aldosterone system inhibitors

## Abstract

Renin‐angiotensin‐aldosterone system inhibitors (RAASi) are recommended for chronic kidney disease (CKD) patients. In this study, we describe RAASi prescription patterns in the Chronic Kidney Disease Outcomes and Practice Patterns Study (CKDopps) in Brazil, Germany, France, and the United States (US). 5870 patients (mean age 66‐72 years; congestive heart failure [CHF] in 11%‐19%; diabetes in 43%‐54%; serum potassium ≥5 in 20%‐35%) were included. RAASi prescription was more common in Germany (80%) and France (77%) than Brazil (66%) and the United States (52%), where the prevalence of prescription decreases particularly in patients with CKD stage 5. In the multivariable regression model, RAASi prescription was least common in the United States and more common in patients who were younger, had diabetes, hypertension, or less advanced CKD. In conclusion, RAASi prescription patterns vary by country, and by demographic and clinical characteristics. RAASi appear to be underused, even among patients with strong class‐specific recommendations. Although the reasons for this variation could not be fully identified in this cross‐sectional observation, our data indicate that the risk of hyperkalemia may contribute to the underuse of this class of agents in moderate to advanced CKD.

## 1 INTRODUCTION

Given the high prevalence of diabetes, proteinuria, and congestive heart failure (CHF) in the chronic kidney disease (CKD) population, clinical practice guidelines support the prescription of renin‐angiotensin‐aldosterone system inhibitors (RAASi) for most patients with CKD as a strategy to limit CKD progression and manage cardiac comorbidities.^1^, ^2^, ^3^ The prescription pattern of RAASi in patients with CKD is still unknown, particularly in general nephrology practice in a “real‐world setting.”

Decreasing renal function and the associated interference with potassium (K) excretion is a major cause for potassium elevation. In clinical practice, however, the development of hyperkalemia is usually the result of a combination of factors superimposed on renal dysfunction, such as diabetes mellitus with high glucose levels or hyporeninemic hypoaldosteronism and advanced stages of heart failure with accompanying reductions in renal perfusion.^4^ Additionally, serum potassium is influenced by the use of medications with hyperkalemic effects, such as angiotensin‐converting enzyme inhibitors (ACEIs), angiotensin receptor blockers (ARBs), mineralocorticoid‐receptor‐antagonists (MRA), and medications that can lead a reduction in serum potassium level, such as diuretics, bicarbonate, and potassium binders.^5^

As such, motivating hypotheses for this analysis are that the prescription of RAASi is relatively low in some patients with CKD, in contrast to practice guidelines and even in real‐life studies based in academic practices. The main objectives of this study were as follows: (a) to describe RAASi use in the Chronic Kidney Disease Outcomes and Practice Patterns Study (CKDopps) overall and by country; by patient characteristics, including CKD stage, level of proteinuria, serum potassium level, diabetic/heart failure status; by use of other medications such as diuretics; and by clinic‐level variation; and (b) to explore patient characteristics associated with RAASi use.

## 2 METHODS

### 2.1 Study design and analytic cohort selection

We conducted a cross‐sectional analysis of data from the CKDopps. CKDopps is an ongoing international prospective cohort study of non‐dialysis patients with estimated glomerular filtration rate (eGFR) <60 mL/min/1.73 m^2^. Participants were sequentially or randomly selected from national samples of nephrologist‐run CKD clinics in Brazil, France, Germany, Japan, and the United States (US). The CKDopps study design and protocol details have been published previously.^6^ The ratio of enrolled patients with eGFR < 30 mL/min/1.73 m^2^ compared with patients with eGFR of 30‐ < 60 mL/min/1.73 m^2^ was 2:1 in Brazil and US clinics; 3:1 in German clinics; and 1:1 in French clinics. At study enrollment, patient demographics and comorbidities are captured, as well as laboratory data up to 6 months prior to enrollment, if available. Routine laboratory data are collected during longitudinal follow‐up based upon how often these data are measured for clinical care, up to a monthly frequency. Medication‐related data are also collected longitudinally throughout study follow‐up. In Brazil, France, and the United States, clinical data are captured via medical record abstraction, while in Germany, data are captured from electronic health records (EHRs) after data review performed by a study coordinator to assess data quality. The study was conducted with adherence to the Declaration of Helsinki and received research ethics board approval (study number: 14004‐05). All patients provided written informed consent before enrollment.

These analyses utilized data from Brazil, France, Germany, and the United States. Data from Japan were not yet available at the time of analysis. In the four participating countries, a total of 7590 patients from national samples of 127 nephrology clinics had enrolled as of September 3, 2018; 430 patients were excluded because the absence of baseline demographic characteristics and medical history data; 492 patients were excluded from the current analyses because of no serum potassium data; and an additional 798 patients were excluded for no medication data, yielding a final analytic cohort of 5870 patients.

### 2.2 Data and statistical analyses

Baseline cross‐sectional data were used for the analyses. Albuminuria was defined and classified into three categories according to Kidney Disease: Improving Global Outcomes (KDIGO) 2012 guidelines—normal or mildly increased (A1, <30 mg/g), moderately increased (A2, 30‐300 mg/g), and severely increased (A3, >300 mg/g), using spot or 24‐hour urine albumin or protein‐to‐creatinine ratio. Comorbidity data were supplemented with medication use and laboratories in France (eg, HbA1c and blood glucose for diabetes) and determined based on International Classification of Diseases (ICD‐10) codes in Germany. Standard descriptive statistics (means and standard deviations [SDs] or medians and interquartile ranges [IQRs] for continuous variables and frequencies for categorical variables) were used to report cohort characteristics by RAASi use at CKDopps entry and country. Serum potassium and RAASi practice patterns were compared across CKD stages and countries, as well as by clinical indication for RAASi use. To identify patient characteristics and other treatments associated with RAASi use, multivariable modified Poisson regression sequential models with sandwich variance estimator were used to estimate RAASi prevalence ratios (PR). All statistical analyses were conducted using SAS, version 9.4 (SAS Institute Inc).

## 3 RESULTS

**Figure 1:**
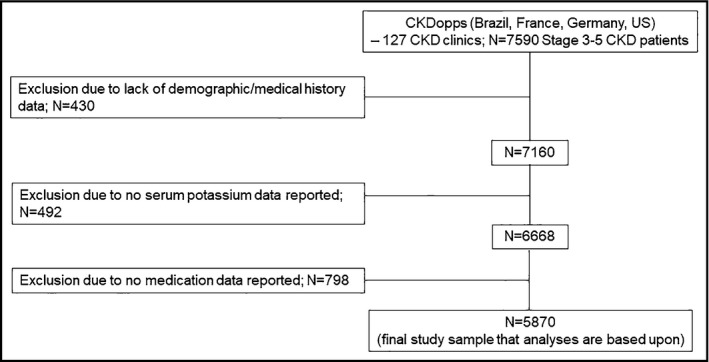
Study flowchart

After the application of inclusion and exclusion criteria, 5870 participants were included in the analysis (Figure 1). Patient characteristics by country and RAASi prescription status are displayed in Table 1. Mean age was 66 years in Brazil, 67 years in France, 72 years in Germany, and 68 years in the United States. The percentage with CKD Stage 4 was 53% in Brazil, 42% in France, 74% in Germany, and 57% in the United States. The most frequently observed comorbidities were hypertension (92% in Brazil, 91% in France, 86% in Germany, and 93% in the United States) followed by diabetes (46% in Brazil, 43% in France and in Germany, and 54% in the United States). The proportion of patients with CHF was 19% in Brazil, 13% in France, 11% in Germany, and 16% in the United States. Among patients with albuminuria measurements, 49% in Brazil, 73% in France, 66% in Germany, and 70% in the United States had moderately to severely increased albuminuria.

**Table 1:** Patient characteristics, by country and RAASi prescription status

	Brazil			France			Germany			US		
	Prescribed RAASi^e^		All	Prescribed RAASi^e^		All	Prescribed RAASi^e^		All	Prescribed RAASi^e^		All
	Yes	No		Yes	No		Yes	No		Yes	No	
Patients, N (%)	340 (66%)	175 (34%)	515	2272 (77%)	678 (23%)	2950	1092 (80%)	276 (20%)	1368	536 (52%)	501 (48%)	1037
Demographics												
Age, years	65.3 (14.8)	68.1 (14.7)	66.2 (14.8)	66.5 (12.7)	68.8 (13.0)	67.0 (12.8)	72.0 (11.8)	71.9 (13.8)	72.0 (12.2)	67.3 (13.0)	68.5 (13.8)	67.9 (13.4)
Black^a^ race, %	27	39	31	3	2	3	‐	‐	‐	22	23	23
Female sex, %	50	44	48	33	41	35	42	46	43	49	48	48
CKD stage, %												
3a	9	2	7	16	16	16	10	7	9	9	7	8
3b	23	15	20	39	36	38	17	18	17	26	17	21
4	55	48	53	42	43	42	73	76	74	57	57	57
5	12	34	20	4	5	4	‐	‐	‐	9	20	14
Comorbidities, %												
Cerebrovascular disease	12	13	13	12	13	12	4	7	4	12	12	12
Coronary artery disease	28	21	25	25	26	25	28	28	28	28	31	29
Congestive heart failure	21	16	19	12	18	13	11	10	11	14	19	16
Diabetes	48	43	46	46	35	43	44	38	43	56	51	54
Hypertension^b^	93	89	92	95	78	91	88	79	86	96	90	93
Peripheral vascular disease	27	18	24	20	19	20	11	13	12	13	17	15
Other cardiovascular disease	14	19	16	26	34	28	14	14	14	19	21	20
Vital sign and laboratories												
SBP, mm Hg	134 (21)	131 (21)	133 (21)	143 (21)	142 (20)	142 (20)	139 (19)	137 (20)	138 (19)	136 (21)	138 (21)	137 (21)
Serum potassium, mEq/L	4.76 (0.59)	4.72 (0.63)	4.75 (0.60)	4.58 (0.50)	4.41 (0.50)	4.54 (0.51)	4.62 (0.64)	4.50 (0.65)	4.60 (0.65)	4.50 (0.55)	4.49 (0.60)	4.50 (0.58)
<3.5	1	2	1	1	3	2	3	4	3	3	3	3
3.5‐<4.5	29	33	31	39	53	43	39	46	40	45	44	44
4.5‐<5.0	33	33	33	37	31	36	30	29	30	34	30	32
5.0‐<5.5	23	20	22	18	11	16	20	15	19	13	19	15
5.5‐<6.0	12	7	10	4	2	3	6	4	6	5	4	4
6.0+	2	5	3	1	1	1	2	3	2	1	1	1
Serum bicarbonate, mEq/L	24.4 (4.3)	24.2 (5.7)	24.3 (5.1)	24.7 (3.4)	25.2 (3.4)	24.8 (3.4)	24.7 (3.8)	24.9 (4.2)	24.8 (3.9)	24.7 (3.8)	24.1 (4.1)	24.4 (3.9)
Albuminuria or equivalent^c^												
Normal to mildly increased	50	53	51	26	29	27	35	32	34	32	29	30
Moderately increased	20	22	20	30	38	32	31	33	31	24	19	22
Severely increased	30	25	29	44	33	41	35	35	35	44	52	48
Prescriptions, % and dietary advice												
Diuretic	78	72	76	57	41	53	73	58	70	69	55	52
Loop	52	60	55	37	38	37	67	55	65	50	47	62
Other	39	15	31	27	4	22	16	13	15	30	13	49
Potassium‐binding resins	0	0	0	14	10	13	5	9	6	0.2	2	22
Sodium bicarbonate	7	12	8	2	4	3	23	26	24	11	17	14
Dietary potassium restriction^d^												
Yes	35	40	37	47	46	47	‐	‐	‐	34	35	34
No	65	60	63	53	54	53	‐	‐	‐	66	65	66

Abbreviations: ACEi, angiotensin‐converting enzyme inhibitors; ARB, angiotensin receptor blocker; CKD, chronic kidney disease; KDIGO, Kidney Disease: Improving Global Outcomes; RAASi, renin‐angiotensin‐aldosterone system inhibitors; SBP, systolic blood pressure; US, United States.

a Includes mulatto in Brazil and patients with parents from Sub‐Saharan Africa or West Indies in France.

b Includes patients who received anti‐hypertensive treatment in French data.

c Thresholds from KDIGO 2012 guidelines: normal or mildly increased (<30mg/g); moderately increased (30‐300 mg/g); severely increased (>300 mg/g); % of missing data is 30% in Brazil, 11% in France, 44% in Germany, and 38% in the United States.

d Among patients with valid answer to the question: During the last 3 mo, did your health care team recommend changes to potassium in your diet? (Brazil/US); Is there a recommendation to pay attention to your consumption of potassium? (France).

e Includes ACEi, ARB, direct renin inhibitors, and aldosterone receptor antagonists.

**Figure 2:**
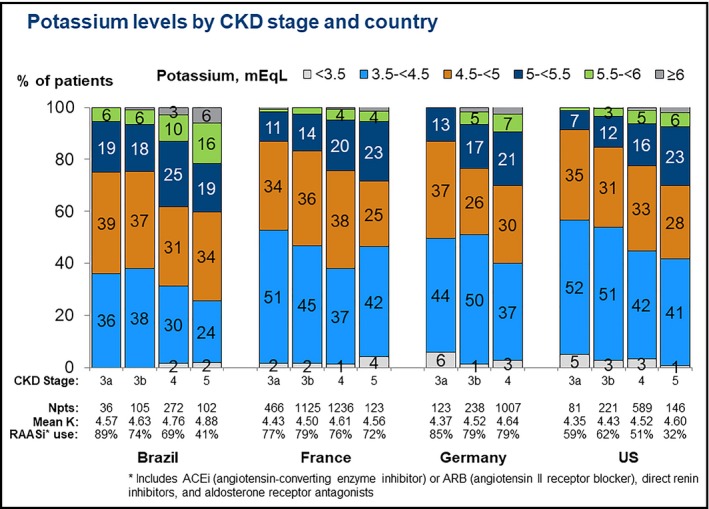
Potassium levels by CKD stage and country. *Includes ACEi (angiotensin‐converting enzyme inhibitor) or ARB (angiotensin II receptor blocker), direct renin inhibitors, and aldosterone receptor antagonists

**Figure 3:**
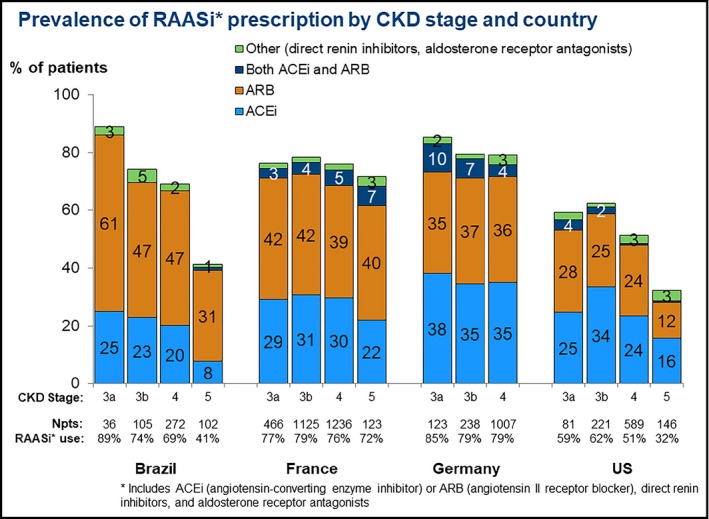
Prevalence of RAASi* prescription by CKD stage and country. *Includes ACEi (angiotensin‐converting enzyme inhibitor) or ARB (angiotensin II receptor blocker), direct renin inhibitors, and aldosterone receptor antagonists

The mean serum potassium level was higher in Brazil (4.75 mEq/L) than in France (4.54 mEq/L), Germany (4.60 mEq/L), and the United States (4.50 mEq/L) (Table 1). Serum potassium levels, by country and by CKD stage, are presented in Figure 2. Serum potassium levels were higher in more advanced stages of CKD. RAASi prescription was more common in Germany (80%) and France (77%) than Brazil (66%) and the United States (52%) (Table 1). The prevalence of RAASi prescriptions, by country and by CKD stage, is shown in Figure 3. RAASi prescription was less common with increasing CKD stage in Brazil and the United States, but no noticeable difference by CKD stage was observed in France and Germany. The type of RAASi prescribed varied by countries, with ARBs more frequently prescribed in Brazil and France than in Germany and the United States. The median prevalence of RAASi prescription within each clinic ranged from 68% in Brazil, 77% in France, 81% in Germany, and 52% in the United States (Figure S1). The between‐clinic variability in prevalence of RAASi prescription within each country was lower in Brazil (IQR: 7%) compared with France (IQR: 14%), Germany (IQR: 14%), and the United States (IQR: 13%).

**Figure 4:**
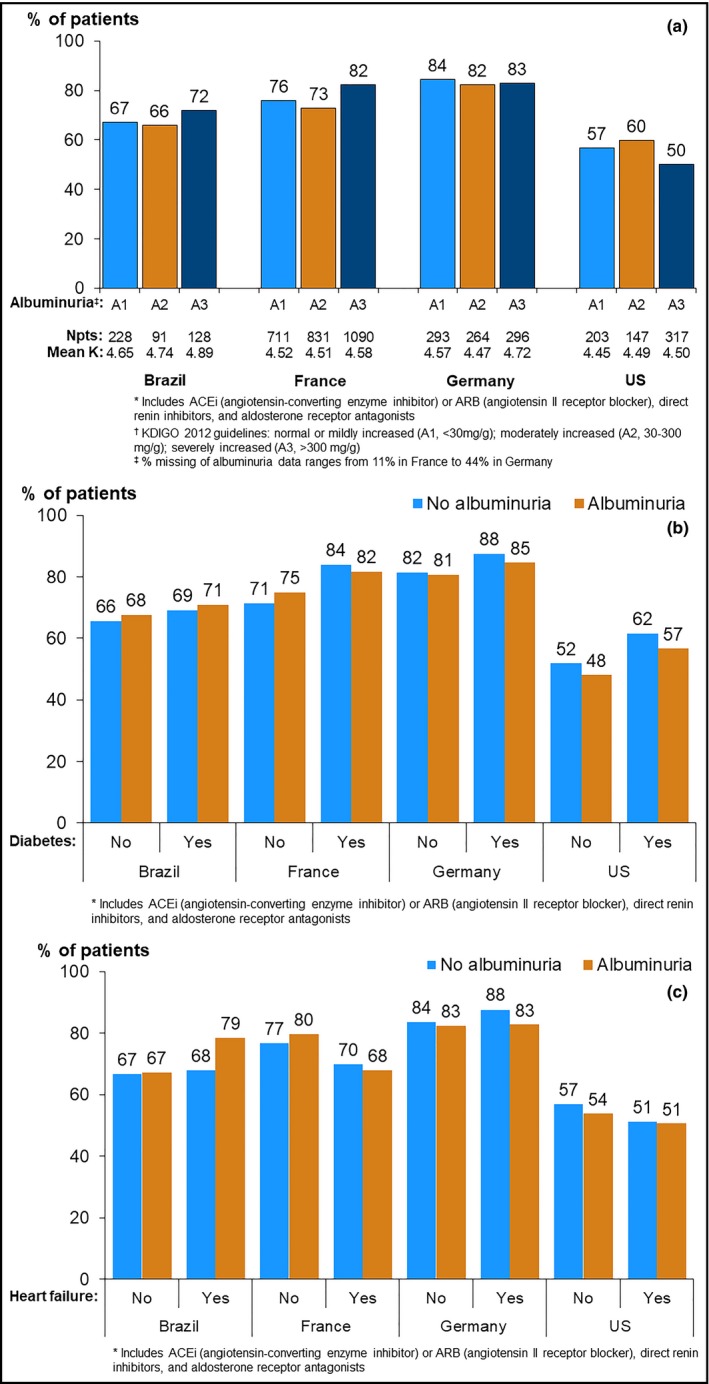
Prevalence of RAASi* prescription, by indication for use and country. A, by level of albuminuria/proteinuria†. *Includes ACEi (angiotensin‐converting enzyme inhibitor) or ARB (angiotensin II receptor blocker), direct renin inhibitors, and aldosterone receptor antagonists. †KDIGO 2012 guidelines: normal or mildly increased (A1, <30 mg/g); moderately increased (A2, 30‐300 mg/g); severely increased (A3, >300 mg/g). ‡% Missing of albuminuria data ranges from 11% in France to 44% in Germany. B, by diabetes status and by albuminuria status. *Includes ACEi (angiotensin‐converting enzyme inhibitor) or ARB (angiotensin II receptor blocker), direct renin inhibitors, and aldosterone receptor antagonists. C, by congestive heart failure status and by albuminuria status. *Includes ACEi (angiotensin‐converting enzyme inhibitor) or ARB (angiotensin II receptor blocker), direct renin inhibitors, and aldosterone receptor antagonists

Patients prescribed RAASi were younger in all countries except Germany, had less advanced CKD, had greater prevalence of diabetes and hypertension, had higher serum potassium level, and more frequently were prescribed diuretics (Table 1). According to the patient self‐administered questionnaire, more than 50% of patients reported that the health care team did not recommend any change in potassium intake for the last 3 months prior to enrollment, despite the prescription of RAASi. The prevalence of RAASi prescription by indication for use is shown in Figure 4. RAASi prescription was more frequent in patients with albuminuria in Brazil and France, but similar despite the presence of albuminuria in other countries (Figure 4A). The prevalence of RAASi prescription was higher among those with diabetes than those without diabetes, despite the presence of albuminuria in France and the United States, but there was no noticeable difference in other countries (Figure 4B). Patients with CHF and albuminuria had higher prevalence of RAASi prescriptions in Brazil, but this was not observed in other countries (Figure 4C).

In the multivariable regression model (Table 2), RAASi prescription was least common in the United States, and more common in patients who were younger, or had diabetes or hypertension. However, the presence of other comorbidities, including CHF, demonstrated no difference in RAASi prescription. Compared with the patients with CKD Stage 3b, the adjusted prevalence of RAASi prescription was similar for CKD Stages 3a and 4, but about 30% lower for patients with CKD Stage 5 (PR = 0.70; 95% CI = 0.61, 0.80). Patients with hypertension (52% higher) or diabetes (8% higher) were more likely to be prescribed RAASi than patients without those comorbidities.

**Table 2:** Prevalence ratios for RAASi^a^ prescription from modified Poisson models with different levels of adjustment

	Model 1	Model 2	Model 3
Country (vs US)			
Brazil	1.27 (1.09,1.49)	1.29 (1.12,1.49)	1.29 (1.12,1.48)
France	1.49 (1.32,1.68)	1.41 (1.25,1.58)	1.44 (1.29,1.62)
Germany	1.53 (1.36,1.73)	1.48 (1.31,1.66)	1.51 (1.34,1.69)
Demographics			
Age, per 10 y		0.97 (0.96,0.99)	0.96 (0.94,0.97)
Female sex (vs male)		0.96 (0.93,0.99)	0.96 (0.93,0.99)
CKD stage (vs stage 3b)			
3a		1.00 (0.94,1.06)	1.00 (0.95,1.05)
4		0.96 (0.92,1.00)	0.96 (0.92,1.00)
5		0.70 (0.61,0.81)	0.70 (0.61,0.80)
Comorbidities (yes vs no)			
Coronary artery disease			0.99 (0.96,1.02)
Congestive heart failure			0.94 (0.89,1.00)
Diabetes			1.08 (1.04,1.12)
Hypertension			1.52 (1.38,1.68)

Abbreviations: ACEi, angiotensin‐converting enzyme inhibitors; ARB, angiotensin receptor blocker; CKD, chronic kidney disease; RAASi, renin‐angiotensin‐aldosterone system inhibitors; US, United States.

a Includes ACEi, ARB, direct renin inhibitors, and aldosterone receptor antagonists.

## 4 DISCUSSION

Current guidelines support the prescription of RAASi for CKD patients, particularly those with diabetes, proteinuria, or heart failure. The results of this multinational study indicate that RAASi are generally underused in advanced CKD, most strikingly in the United States. RAASi prescriptions were more frequent with the presence of diabetes and hypertension comorbidities and less frequent with renal dysfunction, particularly at advanced CKD stages.

There were significant differences in RAASi prescription patterns across the different countries captured in this study, with a consistently higher RAASi prescription in France and Germany compared with the United States and Brazil; these differences were particularly visible in more advanced stages of CKD. These findings contrast with the analysis of the Ramipril Efficacy in Nephropathy (REIN) trial, which shows that the benefit of RAASi in reducing the progression of CKD is not dependent of the degree of kidney dysfunction at the moment when treatment is introduced.^7^ In contrast to current recommendations, RAASi was prescribed in a limited number of individuals of this high‐risk population, especially in the United States and Brazil, even among patients with strong class‐specific recommendations, including albuminuria, diabetes, or heart failure.^1^, ^2^, ^3^^.^ In the present study, patients prescribed a RAASi were younger, had less advanced CKD, and had more comorbidities. No major differences were observed in laboratory values of patients prescribed RAASi compared with those who were not.

Previous studies have shown a variation in RAASi prescription patterns, depending on the setting of patient care. A subgroup with eGFR < 60 mL/min enrolled in the Chronic Renal Insufficiency Cohort (CRIC) study showed that patients with prior contact with a nephrologist were more likely (75%) to receive ACEi/ARB compared with those followed up in primary care (67%). It is noticeable that the prescription pattern of ACE/ARBs in this American academic cohort^8^ shows distinct results compared with the general nephrology practice in a broader sample captured in the present study, which is comparable with the findings of the most recent US Renal Data System report.^9^

While hyperkalemia is only present in 2% of patients treated with RAASi due to hypertension with apparently normal kidney function, the incidence of hyperkalemia in patients with CKD is substantially higher.^10^ In the Irbesartan Diabetic Nephropathy Trial (IDNT) of patients with type 2 diabetes and diabetic nephropathy, the incidence of hyperkalemia, as defined by a serum potassium >6.0 mmol/L, was 18.6%.^11^ Previous studies identified CKD as an important risk factor for hyperkalemia. Interestingly, in patients with eGFR < 30 mL/min, the use of RAAS inhibitors was associated with an increased risk for hyperkalemia, and diuretics with an increased risk for hypokalemia.^12^ In our study, hyperkalemia varied across different countries and particularly CKD stages. As expected, mean serum potassium increases as GFR declines, as does the prevalence of hyperkalemia, as defined by serum potassium higher than 5.5 mEq/L. Hyperkalemia was more common in Brazil than the United States and Germany; several measures used to control hyperkalemia, such as prescription of diuretics, potassium‐binding resins, and bicarbonate supplementation, as well as dietary advice varied across countries, were uncommon. This finding contrasts with the importance of RAASi use and the high risk of hyperkalemia in this study's patient population.

Among medications studied, only the concomitant prescription of diuretics was associated with higher prevalence of RAASi prescription. Diuretics are commonly prescribed in the CKD population, particularly in moderate to advanced CKD for the treatment of hypertension and heart failure, but also as a strategy to control hyperkalemia.^13^ The kaliuretic effects of these drugs may justify the higher prevalence of RAASi prescription in patients prescribed diuretics, since the increase of potassium excretion may reduce the potential of hyperkalemic events.

Given the high‐level evidence in support of RAASi use in CKD patients, emphasis on ways to increase RAASi use is urgently warranted. In this context, the much lower use of RAASi in the United States and Brazil than in Germany and France is striking, and potentially instructive. Higher RAASi use in Germany may be in part due to the substantially greater use of oral bicarbonate and potassium‐binding resins reported in this analysis. Additional study is warranted to understand the effectiveness of such pharmacological interventions in the real‐world setting, including longitudinal follow‐up in CKDopps, other observational studies, and pragmatic interventional studies. The analysis of the impact of the new generation of potassium binders (patiromer and zirconium, which have a better tolerability profile for chronic use) on RAASi prescription, and the potential role of these drugs in reducing the comorbidity burden in CKD, remains to be investigated.^10^

Systems of care likely also influence RAASi use. Even assuming US nephrologists’ intentions to prescribe RAASi to as many CKD patients as possible, disjointed models of care may limit achieving this goal. In the primary care clinic, emergency room, or at hospital discharge, decisions may be made without nephrologist consultation to discontinue RAASi, or to lower dose, in the face of moderate hyperkalemia or small increases in serum creatinine levels. In this context, better coordination of care seems essential to improving RAASi use, as well as other care and outcomes, for these medically challenging patients. Solutions will be undoubtedly complex and may require systems of accountable care, EHRs that span providers,^14^, ^15^ and focused implementation science.^16^

The cross‐sectional design of the analysis has some limitations in the interpretation of these analyses, since it does not allow for analysis of clinical decision‐making, particularly related to events that motivated changes in prescription prior to the inclusion of patients in this study, for example, discontinuation, changes from dual to single therapy, and/or dose reduction that may occur in response to hyperkalemia. Further longitudinal investigation about the change in dose or change in prescription regimens in response to serum potassium levels is needed. Another limitation of the study is that the list of medications captured in the data analysis was limited due to the low prevalence of prescription, such as calcineurin inhibitors, sulfamethoxazole‐trimethoprim, and non‐steroidal anti‐inflammatory agents.

RAASi prescription patterns in CKD vary by country, and by demographic and clinical characteristics. RAASi appear to be underused, especially in the United States, where only half were prescribed a RAASi, even among patients with strong class‐specific recommendations, including albuminuria, diabetes, or heart failure. Higher potassium levels are associated with significantly higher RAASi prescription, before and after adjusting for covariates, an association which is likely to reflect the hyperkalemic effect of RAASi.

In conclusion, it is plausible that RAASi use may be less common in patients at higher risk of hyperkalemia; however, in cross‐sectional analysis, this association may be confounded by: (a) RAASi discontinuation previous to the period of observation; and (b) introduction of anti‐hyperkalemia measures (such as dose reduction, dietary advice, and potassium binder agents) not captured in the observation period. Therefore, prospective studies will need to address whether anti‐hyperkalemia measures may allow greater RAASi use and potentially improve renal and cardiovascular outcomes in patients with moderate to advanced CKD.

## CONFLICT OF INTEREST

Roberto Pecoits‐Filho serves on advisory boards and/or speaks at scientific meetings for Janssen, AstraZeneca, Novartis, Akebia, Baxter Healthcare, and Fresenius Medical Care. He received research grants from Baxter Healthcare, and Fresenius Medical Care. All relationships are modest. Danilo Fliser reports honoraria: Astra‐Zeneca, Bayer Vital, Boehringer Ingelheim and Roche; expert witness and honoraria: Amgen and Vifor FMC. All relationships are modest. Christian Combe received grants for the French CKD‐REIN study from Amgen, Baxter, Fresenius Medical Care, GlaxoSmithKline (GSK), Lilly France, Merck Sharp & Dohme‐Chibret (MSD France), Otsuka Pharmaceutical, and Sanofi‐Genzyme, as well as lecture fees from Fresenius and Amgen. All relationships are modest. Benedicte Stengel received grants for the French CKD‐REIN study from Amgen, Baxter, Fresenius Medical Care, GlaxoSmithKline (GSK), Lilly France, Merck Sharp & Dohme‐Chibret (MSD France), Otsuka Pharmaceutical, and Sanofi‐Genzyme, as well as lecture fees from Lilly and MSD. All relationships are modest. Ziad Massy reports grants for the French CKD‐ REIN study and other research projects from Amgen, Baxter, Fresenius Medical Care, GlaxoSmithKline, Merck Sharp and Dohme‐Chibret, Sanofi‐Genzyme, Lilly, Otsuka, as well as fees and grants to charities from Amgen, Bayer, and Sanofi‐Genzyme. These sources of funding are not necessarily related to the content of the present manuscript. All relationships are significant. All other authors declare “none.”

## AUTHOR CONTRIBUTIONS

Per the International Committee of Medical Journal Editors (ICMJE) criteria, each author contributed important intellectual content during manuscript drafting and revision and accepts accountability for the overall work. Dr Pecoits‐Filho had full access to all data in the study and takes responsibility for its integrity and the data analysis.

## References

[1] Kidney Disease: Improving Global Outcomes (KDIGO) Blood Pressure Workgroup . Clinical practice guideline for the management of blood pressure in chronic kidney disease. Kidney Int. 2012;2(5):337‐414.

[2] Skolnik N , Johnson EL . Clinical guidelines: ADA 2017 standards of medical care in diabetes. Family Practice News 2017. https://www.mdedge.com/familypracticenews/article/130408/diabetes/clinical-guidelines-ada-2017-standards-medical-care. Accessed 7/12/18.

[3] Yancy CW , Jessup M , Bozkurt B , et al. 2016 ACC/AHA/HFSA focused update on new pharmacological therapy for heart failure: An update of the 2013 ACCF/AHA guideline for the management of heart failure: a report of the American college of cardiology/American heart association task force on clinical practice guidelines and the heart failure society of America. Circulation. 2016;134:e282‐e293. 27208050 10.1161/CIR.0000000000000435

[4] Weir MR , Rolfe M . Potassium homeostatis and renin‐angiotensin‐aldosterone system inhibitors. Clin J Am Soc Nephrol. 2010;5(3):531‐548. 20150448 10.2215/CJN.07821109

[5] Chang AR , Sang Y , Leddy J , et al. Antihypertensive medications and the prevalence of hyperkalemia in a large health system. Hypertension. 2016;67(6):1181‐1188. 27067721 10.1161/HYPERTENSIONAHA.116.07363 PMC4865437

[6] Mariani L , Stengel B , Combe C , et al. The CKD outcomes and practice patterns study (CKDopps): rationale and methods. Am J Kidney Dis. 2016;68(3):402‐413. 27113505 10.1053/j.ajkd.2016.03.414

[7] Ruggenenti P , Perna A , Remuzzi G . inhibitors to prevent end‐stage renal disease: when to start and why possibly never to stop: a post hoc analysis of the REIN trial results. Ramipril Efficacy in Nephropathy. J Am Soc Nephrol. 2001;12:2832‐2837. 11729254 10.1681/ASN.V12122832

[8] Ricardo AC , Roy JA , Tao K , et al. CRIC study investigators. Influence of nephrologist care on management and outcomes in adults with chronic kidney disease. J Gen Intern Med. 2016;31(1):22‐29. 26138006 10.1007/s11606-015-3452-x PMC4700009

[9] Saran R , Robinson B , Abbott KC et al. US Renal Data System 2017 Annual Data Report: epidemiology of kidney disease in the United States. Am J Kidney Dis. 2018;71(3 suppl 1):S1‐S672. 10.1053/j.ajkd.2018.01.002 PMC6593155 29477157

[10] Georgianos PI , Agarwal R . Revisiting RAAS blockade in CKD with newer potassium‐binding drugs. Kidney Int. 2018;93:325‐334. 29276100 10.1016/j.kint.2017.08.038 PMC5794635

[11] Lewis EJ , Hunsicker LG , Bain RP , Rohde RD ; The Collaborative Study Group . The effect of angiotensinconverting‐enzyme inhibition on diabetic nephropathy. N Engl J Med. 1993;329:1456‐1462. 8413456 10.1056/NEJM199311113292004

[12] Kovesdy CP , Matsushita K , Sang Y , et al; for the CKD Prognosis Consortium . Serum potassium and adverse outcomes across the range of kidney function: a CKD Prognosis consortium meta‐analysis. Eur Heart J. 2018;39:1535‐1542. 29554312 10.1093/eurheartj/ehy100 PMC5930249

[13] Palmer BF , Clegg DJ . Treatment of abnormalities of potassium homeostasis in CKD. Adv Chronic Kidney Dis. 2017;24(5):319‐324. 29031359 10.1053/j.ackd.2017.06.001

[14] Epstein M , Reaven NL , Funk SE , McGaughey KJ , Oestreicher N , Knispel J . Evaluation of the treatment gap between clinical guidelines and the utilization of renin‐angiotensin‐aldosterone system inhibitors. Am J Manag Care. 2015;21(11 Suppl):S212‐S220. 26619183

[15] Shafi T , Sozio SM , Luly J , et al; DEcIDE Network Patient Outcomes in End Stage Renal Disease Study Investigators . Antihypertensive medications and risk of death and hospitalizations in US hemodialysis patients: Evidence from a cohort study to inform hypertension treatment practices. Medicine (Baltimore). 2017;96(5):e5924. 28151871 10.1097/MD.0000000000005924 PMC5293434

[16] National Institutes of Health (NIH) . ACHIEVE (aldosterone blockade for health improvement EValuation in end‐stage renal disease) Trial # NCT03020303. 2017 https://clinicaltrials.gov/ct2/show/NCT03020303. Accessed 7/12/18.

